# Real-Time Tracking of Laryngeal Motion via the Surface Depth-Sensing Technique for Radiotherapy in Laryngeal Cancer Patients

**DOI:** 10.3390/bioengineering10080908

**Published:** 2023-07-31

**Authors:** Wan-Ju Lee, Yi-Shing Leu, Jing-Sheng Chen, Kun-Yao Dai, Tien-Chi Hou, Chung-Ting Chang, Chi-Jung Li, Kai-Lung Hua, Yu-Jen Chen

**Affiliations:** 1 Department of Radiation Oncology, MacKay Memorial Hospital, Taipei 104217, Taiwan; lee.5712@mmh.org.tw (W.-J.L.); adaidai99.5705@mmh.org.tw (K.-Y.D.); houtc.5708@mmh.org.tw (T.-C.H.); chijung1979@gmail.com (C.-J.L.); 2 Department of Otorhinolaryngology, MacKay Memorial Hospital, Taipei 104217, Taiwan; lys@mmh.org.tw; 3 Department of Computer Science and Information Engineering, National Taiwan University of Science and Technology, Taipei 106335, Taiwan; m11152004@mail.ntust.edu.tw (J.-S.C.); m11115202@mail.ntust.edu.tw (C.-T.C.); 4 Department Medical Research, MacKay Memorial Hospital, Taipei 104217, Taiwan; 5 Department of Artificial Intelligence and Medical Application, MacKay Junior College of Medicine, Nursing and Management, Taipei 112021, Taiwan; 6 Department of Medical Research, China Medical University Hospital, Taichung 404332, Taiwan

**Keywords:** surface depth sensing, laryngeal motion, radiotherapy, laryngeal cancer

## Abstract

Radiotherapy (RT) is an important modality for laryngeal cancer treatment to preserve laryngeal function. During beam delivery, laryngeal motion remains uncontrollable and may compromise tumor-targeting efficacy. We aimed to examine real-time laryngeal motion by developing a surface depth-sensing technique with preliminary testing during RT-based treatment of patients with laryngeal cancer. A surface depth-sensing (SDS) camera was set up and integrated into RT simulation procedures. By recording the natural swallowing of patients, SDS calculation was performed using the Pose Estimation Model and deep neural network technique. Seven male patients with laryngeal cancer were enrolled in this prospective study. The calculated motion distances of the laryngeal prominence (mean ± standard deviation) were 1.6 ± 0.8 mm, 21.4 ± 5.1 mm, 6.4 ± 3.3 mm, and 22.7 ± 4.9 mm in the left–right, cranio–caudal, and anterior–posterior directions and for the spatial displacement, respectively. The calculated differences in the 3D margins for generating the planning tumor volume by senior physicians with and without SDS data were −0.7 ± 1.0 mm (−18%), 11.3 ± 6.8 mm (235%), and 1.8 ± 2.6 mm (45%) in the left–right, cranio–caudal, and anterior–posterior directions, respectively. The SDS technique developed for detecting laryngeal motion during swallowing may be a practical guide for individualized RT design in the treatment of laryngeal cancer.

## 1. Introduction

Head and neck cancers affect approximately 930,000 people per year and cause 465,000 new deaths annually worldwide [1]. Among head and neck cancers, laryngeal cancer (LC) is prevalent, and it is relatively difficult to preserve the larynx during treatment. Inability to preserve laryngeal function profoundly impairs the patient’s quality of life and remains an unmet medical need in cancer treatment. Radiotherapy (RT) could provide an acceptable outcome which is comparable with surgery in early-stage LC [2,3]. For locally advanced LC and hypopharyngeal cancer, induction chemotherapy followed by concurrent chemoradiotherapy (CCRT) could provide a favorable outcome with laryngeal preservation [4,5]. Taken together, the role of RT in the treatment of LC is important in terms of potential laryngeal preservation.

In the past, RT was planned via a two-dimensional technique which contoured the treatment field according to bony markers. In addition, RT was delivered by cobalt-60irradiation, which took more time compared with the current treatment. The study investigating laryngeal motion by Hemlet et al. revealed the period of swallowing during RT delivery was too short to cause a significant change in the total dose, only an approximately 0.5% decline [6]. As technologies advanced, RT technique evolved to intensity-modulated RT (IMRT) or even volumetric modulated arc therapy, which only took 1 min to deliver per treatment. With the shorter duration of time per treatment, the period of swallowing during RT delivery seemed more important, as it might account for a larger percentage of the total duration of treatment.

Many studies showed hypofractionation RT with 2.25 Gy, 3.12 Gy or 3.28 Gy per fraction improved local control in early-stage LC compared with conventional RT [7,8,9]. In view of the good outcome of hypofractionation RT, many further studies investigated stereotactic body radiation therapy (SBRT) in early-stage laryngeal cancer, which revealed dose-limiting grade 3 chronic toxicities [10,11]. However, still some papers recently studied SBRT in early-stage LC [12,13]. In the era of developing SBRT for early-stage LC, laryngeal motions will become an important issue.

Given that progress in advancing technologies promotes precision and therefore reduces the needed margin for sparing normal tissues, RT is emerging as an important component of multimodality treatment in cancers, including LC. However, laryngeal motion remains an uncontrollable uncertainty during beam delivery, especially in the IMRT era with less generous margins. Laryngeal motion is primarily caused by respiration, internal organ motion, and most importantly, swallowing. In head and neck cancer, the most pronounced organ motion develops during LC treatment [14,15,16]. Many investigations have examined laryngeal motion via video-fluoroscopic swallowing study, 4D computed tomography (CT), cone beam CT, and dynamic magnetic resonance imaging (MRI) [14,15,17,18,19]. Their results suggest that the most significant motion is in the cranio–caudal direction rather than in the anterior–posterior or left–right direction [6,14,17,19]. Collectively, the investigation of laryngeal motion to allow real-time monitoring and possibly gate-targeted RT is a critical clinical issue in the daily practice of RT. Surface-guided RT has been applied in clinical cancer treatment of breast, lung, and other cancers [20,21]. The current optical surface scanning by 3D cameras equipped in surface-guided RT is unable to track random, rapid, and extensive motions, such as motion of the larynx. To monitor the real-time laryngeal motion with a characteristic feature, particularly the moving laryngeal prominence, the detection of differentiation between surface and depth by using a specified camera system may be useful [22].

In our study, we used the surface depth-sensing (SDS) technique for tracking laryngeal motion, with a specific focus on the laryngeal prominence. Our approach aims to develop a method to track the real-time laryngeal motion in a non-invasive, non-labor-consuming, inexpensive and radiation-free manner.

## 2. Materials and Methods

### 2.1. Study Design and Participants

This study was a collaboration with the Department of Computer Science and Information Engineering, National Taiwan University of Science and Technology. Patients diagnosed with LC who were aged between 18 and 80 years old would be enrolled in this prospective study. All of them received conventional radiotherapy as treatment. The performances of the enrolled cases were between grade 0 and 2. In concern of limited laryngeal motion, patients with vocal cord impairment or fixation were ineligible. This study was granted approval by the Institutional Review Board of MacKay Memorial Hospital (#17MMHIS056), and all the patients signed the informed consents accordingly. This study abides by the Declaration of Helsinki.

### 2.2. Simulation, RT Planning, and Beam Delivery

All the patients were laid in the supine position and immobilized by thermoplastic facial masks. After cooling, the masks were cut at the site of the nose for breathing. Isocenters were labeled on the mask using laser markers to ensure reproducibility. Thereafter, the patients underwent contrast-enhanced CT with a thickness of 3 mm per slice.

Six patients with early-stage LC were treated with photon RT (60–66 Gy in 30–36 fractions) to the laryngeal box field. In the CCRT planning of locally advanced LC, gross lesions and lymphadenopathy were treated with 70 Gy, whereas risky lymph node regions were treated with 56–63 Gy. The daily fraction size was 1.8–2 Gy, and RT was delivered 5 days a week. The planning tumor volume (PTV) was 0.3–1 cm extra than the clinical target volume (CTV) to account for organ motion and setup error following international consensus [23]. The RT planning system (Eclipse version 13.0, Varian, CA, USA) was applied accordingly. All the treatments were administered via the IMRT technique using a linear accelerator (Clinac iX, Varian, CA, USA) with 6 MV or 10 MV.

### 2.3. Setup for the SDS Technique

**Figure 1:**
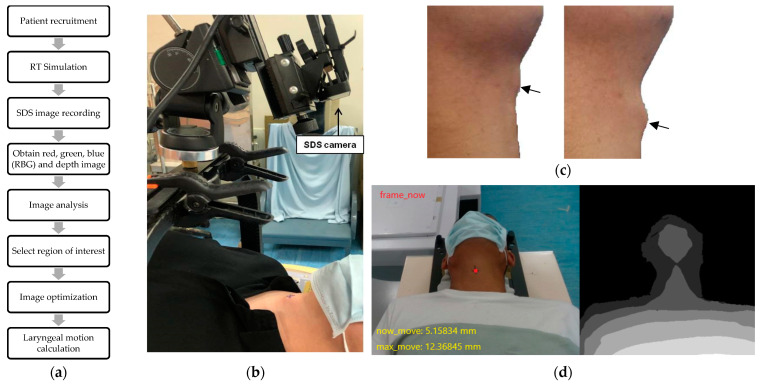
Setup and recording for the SDS technique. (**a**) Experimental workflow of our study. (**b**) Setup for the SDS camera. (**c**) Obtain obvious swallowing motion for three times. The arrows indicate the laryngeal prominence. (**d**) Real-time image under the SDS technique, including red, green, blue (RBG) (**left**) and depth (**right**) images.

The patients were laid in the same position as the simulation without masks. Hard headrests similar to those in the simulation were also used for lifting the patients’ chin up and making the laryngeal motion clear. Prior to initiation of tracking, we made a cross marker at the resting laryngeal prominence as an initial reference point for the image center. An SDS camera (S/N W4VF, CREATIVE, Singapore) was kept in front of the patients, focusing on the cross marker. After ensuring comfortable and acceptable positioning, videotaping using the SDS camera was performed. Thereafter, the patients were asked to swallow three times consecutively. The patients were recorded till the three consecutive swallowing were completed to collect real-time imaging parameters (Figure 1).

### 2.4. Algorithm for the Surface Depth Calculation

#### 2.4.1. Image Optimization

**Figure 2:**
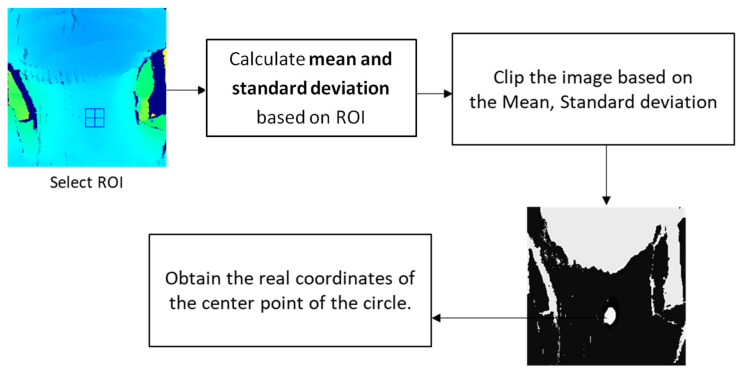
Flowchart of the optimization process.

To enhance the images to emphasize a specific part, the image data were normalized on a section basis rather than treating each pixel in the entire image equally. The regions of interest (ROIs) were selected to compute the mean and standard deviation, and the lower and upper ranges were applied by clipping the characteristic feature in the images. After normalization, the circular area of the laryngeal motion could be observed, and the real coordinates of its center point could be obtained. After that, the highest and lowest points were coordinated, and the distance between the two points was calculated. Figure 2 demonstrates the processing flowchart of the optimization process.

#### 2.4.2. Algorithm for the Surface Depth Calculation

**Figure 3:**
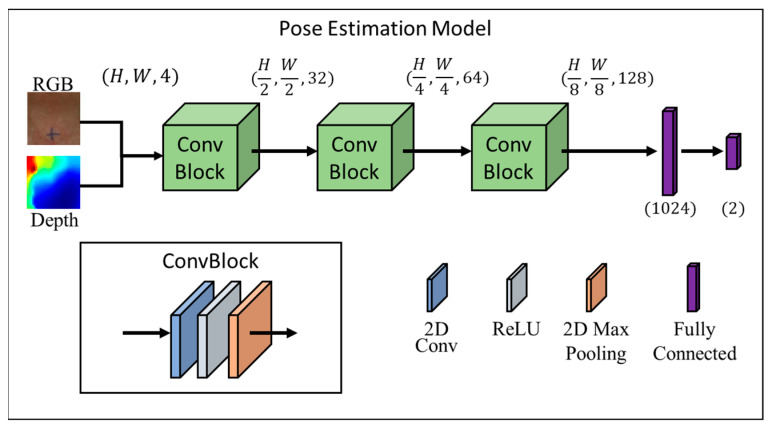
Architecture of our Pose Estimation Model, which takes as the input a fusion of RGB and depth images and outputs the position of the larynx.

The SDS data were calculated via the Pose Estimation Model. The procedure for processing the image data was as follows (Figure 3). The input of the Pose Estimation Model was an RGBD (red, green, blue and depth) image, which was a color image that contained depth information, and the output was the predicted position coordinate in the format of (x,y). For effective and real-time tracking, we used ConvBlock in Figure 1, which is one of the commonly used modules in convolutional neural networks which could effectively extract features from images and reduce the number of parameters [24]. The model consisted of three ConvBlocks. After the three ConvBlocks, the output was flattened into a 1D vector and fed into two fully connected layers for the final prediction. The following would introduce the details of the model, including the factors considered and how the model output was transformed into real-world coordinates.

#### 2.4.3. Preliminary Information

Depth information was crucial for finding the position of the larynx. Therefore, we fused the RGB and depth information together in an H × W × 4 input format. The depth information was used to estimate the 3D position of the larynx, while the RGB information provided color and texture cues that helped to identify the larynx based on its appearance.

#### 2.4.4. ConvBlock

The ConvBlock was a building block that consisted of a convolutional layer followed by a ReLU activation function and a pooling layer. By using multiple ConvBlocks in the model, the model was able to learn increasingly complex and abstract representations of the input data. The first ConvBlock extracted low-level features, such as edges and corners, while the subsequent ConvBlocks built on these low-level features to extract higher-level features, such as shapes and patterns [25]. The ReLU activation function was used after each convolutional layer, which introduced nonlinearity into the model and allowed it to learn more complex patterns [26]. The use of max pooling layers also helped to reduce the spatial dimensions of the feature maps, which reduced the number of parameters in the model and helped to prevent overfitting, which helped the model to generalize well to unseen data while also enabling real-time processing.

#### 2.4.5. Real-World Coordinates

The predicted point from the model needed to be converted into real-world coordinates based on the camera parameters so that it could be accurately targeted during RT. Assuming that the camera’s internal parameters were known and the camera had been calibrated with the external parameters estimated, given a predicted pixel coordinate $\left(x,y\right)$ and corresponding depth value D, the real-world coordinate $\left(X,Y,Z\right)$ could be computed in Equation (1)

(1) $$X=\left(x-c_{x}\right)\times \frac{D}{f_{x}}\;Y=\left(y-c_{y}\right)\times \frac{D}{f_{y}}\;Z=\;D$$

where $f_{x}$ and $f_{y}$ are the camera’s focal lengths, and $c_{x}$ and $c_{y}$ are the principal point coordinates. This formula assumed a small distortion or already corrected distortion, and further distortion correction would be required for more accurate 3D coordinates if the distortion was significant [27].

#### 2.4.6. Implementation Detail

The Computer Vision Annotation Tool (CVAT) was used to manually assign labels to the position of the patient’s larynx [28], and the PyTorch framework was applied to implement our method using [29]. In the following, we will demonstrate the details of the training and validation phases. In the training phase, we set the input size to 254 × 254. The total number of videos in the dataset was 7. We used 5 videos for the training set and the total number of frames was 3576. We trained our network to simply use an L1 loss with 4 mini-batches and optimize it using the Adam optimizer [30] (β1 = 0.9, β2 = 0.99), with a learning rate of 10-3. Our model was trained on a Graphic Processing Unit (GTX 1650, NVIDIA, CA, USA). In the validation phase, we used the remaining 2 videos for the validation set and the total number of frames was 830. We calculated the L1 loss based on the validation set and selected the model with the smallest loss as the final model.

### 2.5. Validation of Reproducibility

To ensure the reproducibility of the SDS detection, each patient received three consecutive measurements by the same researcher with the same setup conditions.

### 2.6. Application of SDS Data to Generate the PTV

To estimate the inter-observer variation, three independent and licensed radiation oncologists were recruited to set up the PTV margins for laryngeal motion for each patient with and without SDS data. Initially, the cancer profile would be provided to the radiation oncologists, who determined the PTV margins for each patient accordingly. Following determining the PTV without SDS data, the SDS data for each patient were provided. The radiation oncologists could modify their PTV based on the SDS data. The difference between the PTV margin with or without the SDS technique assisting was calculated and a paired *t*-test was performed to assess the significance.

### 2.7. Statistical Analysis

Descriptive statistical analysis was performed using SPSS software (IBM Corp. Released 2019. IBM SPSS Statistics for Macintosh, Version 24.0. Armonk, NY, USA). Continuous variables were analyzed via a paired *t*-test. A *p*-value less than 0.05 was considered to be statistically significant.

## 3. Results

### 3.1. Subject Enrollment

Six patients with early LC and one patient with locally advanced LC, which was clinical T2N2bM0, stage IVA, were enrolled in this prospective study. The T2 stage was only subglottic extension without vocal cord impairment or fixation. Patient characteristics are shown in Table 1. These patients received definitive RT or CCRT to preserve the larynx.

### 3.2. Calculation of Laryngeal Motion

The calculated motion distances of the laryngeal prominence were 1.6 ± 0.8 mm, 21.4 ± 5.1 mm, 6.4 ± 3.3 mm, and 22.7 ± 4.9 mm in the left–right, cranio–caudal, and anterior–posterior directions and for the spatial displacement, respectively. Detailed data are shown in Table 2.

### 3.3. PTV Margin with and without SDS

**Figure 4:**
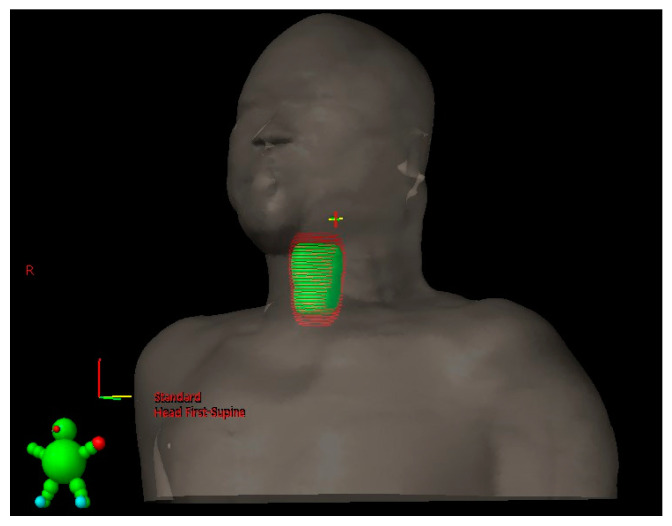
The difference in the PTV volumes with and without SDS assistance. The green object indicates the volume of the PTV without the assistance of the SDS technique, and the red one reveals the PTV with the assistance of the SDS technique.

As demonstrated in Table 3, the PTV margins generated from the CTV were defined as 3.9 ± 1.0 mm, 4.8 ± 2.7 mm, and 4.0 ± 1.0 mm in the left–right, cranio–caudal, and anterior–posterior directions, respectively, without SDS data. Intriguingly, the margins with SDS data changed to 3.2 ± 0.6 mm, 16.1 ± 6.4 mm, and 5.8 ± 2.6 mm in the left–right, cranio–caudal, and anterior–posterior directions, respectively. The postulated differences in the 3D margins for generating the PTV by senior physicians with and without SDS data were −0.7 ± 1.0 mm, 11.3 ± 6.8 mm, and 1.8 ± 2.6 mm in the left–right, cranio–caudal, and anterior–posterior directions, respectively. On comparing the difference with the data obtained without SDS, the percentages of the changes in the left–right, cranio–caudal, and anterior–posterior directions, respectively, were noted to be −18% (*p* = 0.005), 235% (*p* < 0.001), and 45% (*p* = 0.004), indicating a significant difference in all directions, especially in the cranio–caudal direction, as shown in Figure 4.

### 3.4. Time of Performance

A total of seven recordings were performed per person. Because of the convenience of the machine setup and checking the image quality in real time, no patient was asked to return for the SDS technique. The average time spent on the video recording was 41 ± 19 s.

### 3.5. Perception of Subjects

All the patients tolerated the procedures. During the SDS technique, no significant procedure-related adverse effects, including pain, anxiety, and ipovlopsychophobia, were documented.

## 4. Discussion

In this study, we developed an SDS technique to detect laryngeal motion. The results demonstrated that spatial laryngeal motion may vary vigorously on an individual basis. This implies that the margin needed for generating the PTV should be individualized to adequately cover the inter- and intra-fractional variations.

In contrast, the results reported using cine MRI revealed that the motion distances in the cranio–caudal and anterior–posterior directions were 7.1 mm and 4.2 mm, respectively [16]. Using surface-guided detection, the motion distances were 5.8 mm above baseline, especially in the longitudinal direction [12]. These two studies were performed by instructing patients to withhold swallowing during the acquisition of motion data. The motion distances from the non-swallowing conditions were significantly smaller than those from our data. However, self-control over swallowing usually is uncomfortable and difficult to obey, and it might be more practical to swallow under command. One video-fluoroscopic swallowing study conducted by Hamlet et al. showed a similar motion distance to our data, which revealed that the larynx moved 2 cm longitudinally and less than 1 cm anteriorly during swallowing, indicating the detection of motion distances under natural swallowing is more vigorous than static measurement, and it might be more feasible for clinical applications [6]. A video-fluoroscopic swallowing study detects 2D motion. In the era of IMRT/IGRT, the detection of the 3D motion distance, such as the results in the present study, is clinically relevant.

Given the progress in advancing technologies, an increasing number of techniques are investigating laryngeal movement. One study conducted by Huynh et al. revealed laryngeal motion can be tracked and gated by the cine MR in the MR-Linac [31]. Another study applied three-dimensional cameras to track 16 markers on the chin and anterior neck to evaluate the extrinsic laryngeal muscle tension and hyperfunction [32]. Zhang et al. designed a wearable swallowing recognition system based on motion and dual photoplethysmography to sense laryngeal movement [33]. Compared with the above-mentioned techniques, the SDS technique is non-labor-consuming and inexpensive because the SDS camera costs only around USD 300.

In our study, the average PTV margin in the cranio–caudal direction increased more than two-fold with the assistance of the SDS technique, and that in the anterior–posterior direction increased by approximately 50%. In contrast, the margin in the left–right direction decreased by 18% with the SDS data. Consequently, the laryngeal motion seems to be underestimated, particularly in the cranio–caudal direction. In contrast, the horizontal movement of the larynx might be overestimated in previous clinical practice. With the aid of the SDS technique, it would be possible to design individualized margins for personalized radiotherapy, as an integral component of precision medicine.

The results of our study showed that the average distance of laryngeal motion during RT delivery was approximately 21 mm in the cranio–caudal direction. However, the three licensed radiation oncologists we recruited only added 16 mm to set up the PTV margins for laryngeal motion with SDS assistance. The reason for the difference might be the toxicities of RT deriving from the large volume of the treatment field. Further study investigated the relation between the route of laryngeal motion and the time factor. With the above-mentioned data, the tracking plot which showed the time period of the laryngeal prominence at a specified distance accounting for the total tracking duration could be finely assessed, exclude the extremum and find the appropriate margin for individuals.

The average SDS tracking time was less than 1 min. Including setup and confirmation of the image, the total SDS technique took approximately 5 min for each patient. Moreover, no patient complained of discomfort during the execution of this technique. As stated above, the results suggest that the SDS tracking technique is an efficient, time-saving, and comfortable approach for clinical applications.

Applying the SDS technique to track laryngeal motion in real time seemed feasible and promising. However, there were some limitations in this prospective study. We found that the surface depth would be influenced by the mandible when the larynx would move upward. Therefore, the maximum margin of laryngeal cranial motion may not be detectable, so the accuracy and reliability at the mandible level would become poorer. Since the major movement path of the larynx was allocated within the ROI, the above limitation might be overcome by further validation of the upmost laryngeal motion using a simulated CT scan and/or cone beam CT images. In addition, the small number size with all male patients is a limitation. In this study, we primarily intended to explore the development of surface depth-sensing technology for evaluating laryngeal movement during swallowing. Due to the lower incidence of female laryngeal cancer, the enrollment of female patients is relatively difficult. This small-scale clinical investigation with male patients is a preliminary proof-of-concept test. Further clinical investigations with large cohorts of female and male patients will be conducted before integrating this technology into daily practice.

The SDS technique is a convenient, non-invasive, non-labor-consuming, inexpensive, and radiation-free method to track laryngeal motion. Radiation oncologists can modify the PTV margin according to the tracking data for individualized RT. In the era of developing SBRT for early-stage laryngeal cancer, laryngeal motions will be a crucial issue [12,13]. From the biological perspective, molecular imaging is a recently promising way to detect tumor microenvironment-responsive contrast agents which obtain a higher signal-to-noise ratio and lower background interference. With the characteristics of tumor microenvironment-responsive contrast agents, molecular imaging is feasible for specific cancer imaging [34]. The combination of SDS technology and molecular imaging for tracking tumor in both physic and biological manners may have potential to be developed. Despite the promising results, future research which aims to expand the study cohort to validate the effectiveness and reproducibility of the SDS technique is needed. Furthermore, the real-time SDS tracking technique might enable possible development of swallowing gating for adaptive RT, although the feasibility still warrants further investigation.

## 5. Conclusions

The SDS technique developed in this study provides a practical and innovative approach for real-time tracking of laryngeal motion during swallowing. By integrating this technique into RT planning, the SDS technique may serve as a practical guide for individualized RT design in the treatment of laryngeal cancer. Future studies with large cohorts of female and male patients and explorations of swallowing gating for adaptive RT will further establish the potential of the SDS technique in the treatment of laryngeal cancer.

## Figures and Tables

**Table 1:** Patient characteristics.

No.	Diagnosis	Stage	Age (Years)	Sex	BMI
1	LC	II	77	Male	23.1
2	LC	I	54	Male	24.5
3	LC	IVA	69	Male	21.2
4	LC	II	75	Male	24.4
5	LC	I	64	Male	21.6
6	LC	I	78	Male	21.9
7	LC	I	52	Male	26.0

LC, laryngeal cancer.

**Table 2:** Parameters of laryngeal motion.

No.	LR (mm)	CC (mm)	AP (mm)	Distance (mm)
1	2.0	16.0	3.0	16.4
2	1.7	29.9	5.6	30.5
3	2.5	24.6	3.9	25.0
4	1.6	16.4	10.5	19.5
5	1.3	17.5	5.5	18.4
6	0.1	23.2	4.6	23.7
7	2.0	22.5	11.5	25.3
Mean ± SD	1.6 ± 0.8	21.4 ± 5.1	6.4 ± 3.3	22.7 ± 4.9

LR, left–right direction; CC, cranio–caudal direction; AP, anterior–posterior direction; SD, standard deviation.

**Table 3:** Difference in the 3D PTV margin with or without the SDS technique.

	LR			CC			AP		
	Without SDS	With SDS	Difference	Without SDS	With SDS	Difference	Without SDS	With SDS	Difference
Mean ± SD (mm)	3.9 ± 1.0	3.2 ± 0.6	−0.7 ± 1.0	4.8 ± 2.7	16.1 ± 6.4	11.3 ± 6.8	4.0 ± 1.0	5.8 ± 2.6	1.8 ± 2.6
Percentage of change			−18%			235%			45%
*p* value			0.005			<0.001			0.004

LR, left–right direction; CC, cranio–caudal direction; AP, anterior–posterior direction; SD, standard deviation.

## Data Availability

The datasets presented in this study are available from the corresponding author on reasonable request.
